# Endoscopic optical diagnosis provides high diagnostic accuracy of esophageal squamous cell carcinoma

**DOI:** 10.1186/1471-230X-14-141

**Published:** 2014-08-09

**Authors:** Kengo Nagai, Ryu Ishihara, Shingo Ishiguro, Takashi Ohta, Hiromitsu Kanzaki, Takeshi Yamashina, Kenji Aoi, Noriko Matsuura, Takashi Ito, Mototsugu Fujii, Sachiko Yamamoto, Noboru Hanaoka, Yoji Takeuchi, Koji Higashino, Noriya Uedo, Hiroyasu Iishi, Masaharu Tatsuta, Yasuhiko Tomita, Takashi Matsunaga

**Affiliations:** 1 Departments of Gastrointestinal Oncology, Osaka Medical Center for Cancer and Cardiovascular Diseases, 3-3, Nakamichi 1-chome, Higashinari-ku, Osaka 537-8511, Japan; 2 PCL Osaka Inc., Osaka, Japan; 3 Department of Gastroenterology, NTT West Osaka Hospital, Osaka, Japan; 4 Department of Gastroenterology and Hepatology, Okayama University Graduate School of Medicine, Okayama, Japan; 5 Departments of Pathology, Osaka Medical Center for Cancer and Cardiovascular Diseases, Osaka, Japan; 6 Departments of Medical Informatics, Osaka Medical Center for Cancer and Cardiovascular Diseases, Osaka, Japan

**Keywords:** Esophageal neoplasms, Esophageal cancer, Optical biopsy, Narrow-band imaging, Endoscopic diagnosis

## Abstract

**Background:**

Recent technological advances have stimulated the development of endoscopic optical biopsy technologies. This study compared the accuracy of endoscopic diagnosis using magnifying narrow-band imaging (NBI) and histologic diagnosis of esophageal squamous lesions.

**Methods:**

Patients at high risk for esophageal squamous cell carcinoma were examined with endoscopy and subsequent biopsy. The lesions diagnosed as cancer on NBI and the lesions diagnosed as cancer on biopsy were resected endoscopically or surgically. Histological diagnoses of resected specimens, the reference standards in this study, were made by a pathologist who was blind to both the endoscopic and biopsy diagnoses. The primary outcome was the accuracy of endoscopic and biopsy diagnosis. A noninferiority trial design with a noninferiority margin of −10% was chosen to investigate the accuracy of endoscopic diagnosis using magnifying NBI.

**Results:**

Between November 2010 and October 2012, a total of 111 lesions in 85 patients were included in the analysis. The accuracy of endoscopic diagnosis and biopsy diagnosis for all lesions was 91.0% (101/111) and 85.6% (95/111), respectively. The difference in diagnostic accuracy was 5.4% (95% confidence interval: −2.9%–13.7%). The accuracy of endoscopic diagnosis and biopsy diagnosis of invasive cancers was 94.9% (74/78) and 84.6% (66/78), respectively. The difference was 10.3% (95% confidence interval: 1.6%–19.0%) for invasive cancers. The lower bound of the 95% confidence interval was above the prestated −10% in both cases.

**Conclusion:**

Noninferiority of endoscopic diagnosis by magnifying NBI to histologic diagnosis by biopsy was established in this study (p = 0.0001).

**Trial registration:**

The study was registered on 9^th^ November 2010 in the University Hospital Medical Network Clinical Trials Registry as number: UMIN000004529.

## Background

Esophageal cancer is the sixth most common cause of cancer-related mortality worldwide [1]. The overall survival of patients with esophageal cancer, regardless of histological type, remains poor. However, a favorable prognosis can be expected if this cancer is detected at an early stage [2-5]. Diagnosis of early esophageal cancers is based on the detection of suspicious lesions and histological evaluation of specimens taken from these suspicious lesions.

Endoscopically or surgically resected specimens with total biopsy of the lesions would provide the most accurate histologic diagnosis and can serve as the reference standard of histologic diagnosis. There are reports of discrepancy between diagnosis based on biopsy specimens and diagnosis based on endoscopically resected specimens, suggesting limited accuracy of biopsy diagnosis [6,7]. A high false-negative rate of biopsy diagnosis of esophageal, gastric, and colon cancers has also been reported [8,9]. Such limitations in the accuracy of biopsy diagnosis may be associated with the sampling process or diagnostic process for small specimens. Taking 3 to 10 biopsy specimens would improve the accuracy of this technique [10-13]. However, multiple biopsies can increase the risk and cost of the procedure and potentially make subsequent endoscopic resection difficult [14-16].

Recent technological advances have stimulated the development of numerous optical methods. These methods allow for accurate evaluation and diagnosis of cancers in vivo and are thus termed optical biopsy techniques. Endoscopic optical biopsy techniques offer noninvasive real-time diagnosis. Some techniques currently being evaluated include optical coherence tomography [17,18], endocytoscopy [19], and narrow-band imaging (NBI) [20]. NBI is an imaging technique that enhances the visualization of mucosal microstructures and microvessels. Previous studies involving NBI and magnification have shown high diagnostic accuracy for esophageal squamous cell carcinoma [21-24]. Although many endoscopic techniques have preliminarily shown high accuracy rates, these technologies are still evolving, and the accuracy of endoscopic diagnosis has not yet been fully investigated. Endoscopic diagnosis has the advantage of providing noninvasive and real-time diagnosis without the additional cost of biopsy. If the accuracy of endoscopic diagnosis is comparable to that of histologic diagnosis of biopsy specimens, endoscopic optical biopsy can be used in some situations. However, few studies have compared the accuracy of endoscopic optical diagnosis with that of histologic biopsy diagnosis.

This study compared the accuracy of endoscopic diagnosis using magnifying NBI versus histologic diagnosis of esophageal squamous lesions. The accuracy was evaluated using lesions diagnosed as cancer on biopsy and lesions endoscopically diagnosed as cancer. Histologic diagnosis of resected specimens served as the reference standard. A noninferiority trial design was adopted under the consideration that a similar or slightly reduced accuracy of endoscopic diagnosis might be accepted because it would be balanced by other benefits such as less invasiveness, less cost, and real-time results.

## Methods

### Patients

The study protocol was approved by the Ethics Committee of the Osaka Medical Center for Cancer and Cardiovascular Diseases. The study was registered in the University Hospital Medical Network Clinical Trials Registry (UMIN-CTR) as number UMIN 000004529. The patient inclusion criteria were the presence of esophageal neoplasia, a history of esophageal cancer treated by endoscopic resection, and current or past head and neck cancer. Patients were excluded if they had undergone previous surgery, chemotherapy, or radiotherapy for esophageal cancer. Patients were also excluded if they had severe reflux esophagitis or an allergy to iodine.

### Endoscopic examinations and biopsies

The endoscopic procedures were carried out using a high-resolution magnifying upper gastrointestinal endoscope (GIF-Q240Z or GIF-FQ260Z; Olympus, Tokyo, Japan) or a high-definition magnifying upper gastrointestinal endoscope (GIF-H260Z; Olympus). The structure-enhancement function of the video processor was set at a level of B8 (strongest enhancement level for microstructures) for NBI observation. A black soft hood (MB-162 for GIF-Q240Z and MB-46 for FQ260Z and GIF-H260Z; Olympus) was mounted on the tip of the endoscope to maintain an adequate distance between the tip of the endoscope zoom lens and the mucosal surface during magnifying observation. Initial routine inspection was carried out with white-light imaging. The surface vascular pattern of the lesion was then observed by magnifying NBI. These procedures were followed by chromoendoscopy with iodine solution.

**Figure 1:**
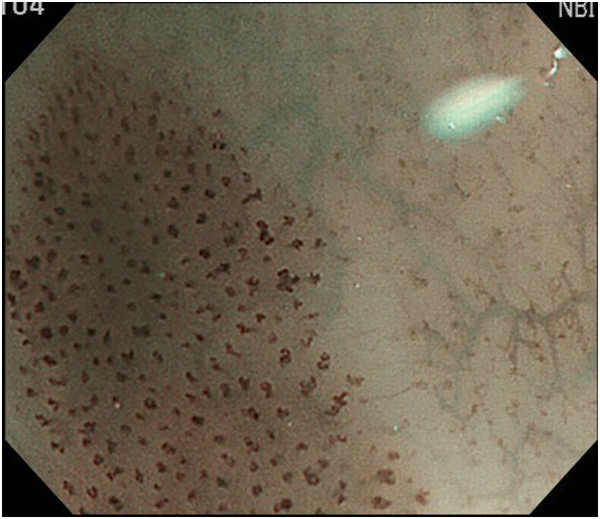
A lesion with well-demarcated brownish change of the epithelium and scattered brown dots.

**Figure 2:**
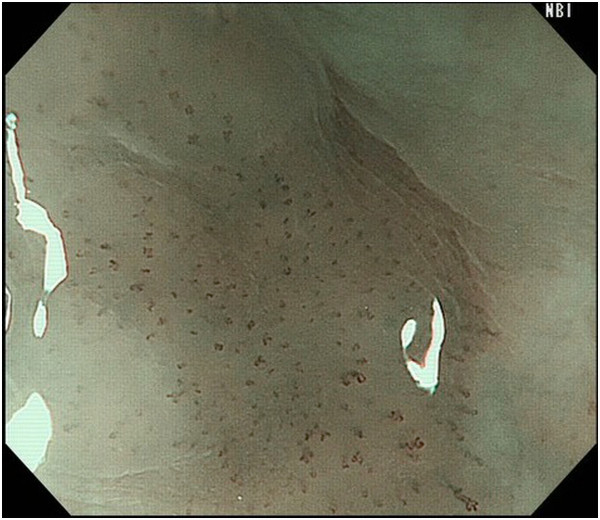
A lesion with obscure brownish change and obscure scattered brown dots.

**Figure 3:**
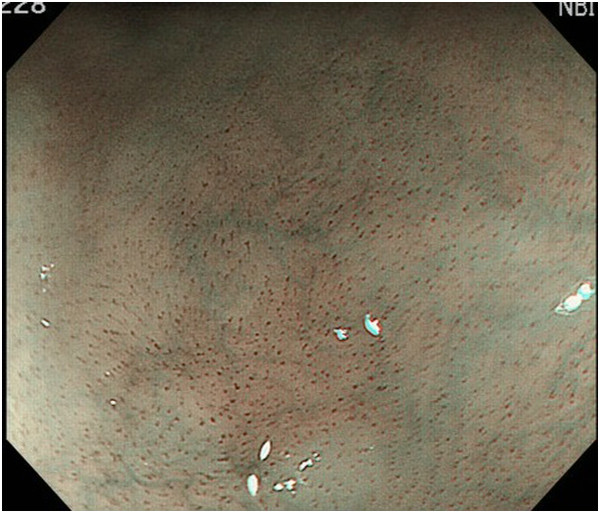
A lesion without any dilated and tortuous vessels.

Endoscopic diagnosis using magnifying NBI was made as follows. Cancer was diagnosed when well-demarcated brownish change of the epithelium and scattered brown dots or dilated, tortuous vessels of various sizes were identified (Figure 1) [24,25]. An undetermined status was assigned when an obscure brownish change or obscure scattered brown dots were present (Figure 2). The absence of cancer was diagnosed when no brownish change or scattered brown dots were present (Figure 3). Biopsy specimens were taken from iodine-unstained lesions or lesions that were diagnosed as cancer or undetermined on NBI. Lesions in the cervical esophagus were excluded from the analysis because endoscopic observation and biopsy of these lesions are usually difficult. Lesions of ≤5 mm were also excluded from the analysis because most of them would likely be removed by biopsy. The endoscopic reports, which included lesion sizes but not endoscopic diagnoses, were sent to the pathologist. Biopsy specimens were embedded in paraffin and subjected to staining with hematoxylin and eosin. Pathologists with special qualifications made histological diagnoses of cancer based on structural and cytological abnormalities.

### Endoscopic resection and histologic assessment

The lesions diagnosed as cancer on NBI and the lesions diagnosed as high-grade intraepithelial neoplasia or cancer on biopsy were resected endoscopically or surgically. Lesions were also resected when they showed an obvious pink color change after iodine staining [26,27]. Resected specimens were embedded in paraffin and subjected to hematoxylin and eosin staining. Another pathologist with special qualifications (S.I.) who was blind to the endoscopic and biopsy diagnoses made the histological diagnoses according to the WHO criteria for the classification of early gastrointestinal neoplasia [28]. Lesions with structural and cytological abnormalities reaching the upper half of the squamous epithelium were diagnosed as cancer in situ, also termed high-grade intraepithelial neoplasia [28]. The lesions were also diagnosed as cancer based on obvious cytological abnormalities of the squamous epithelium, even when the abnormalities were confined to the lower half of the squamous epithelium [29]. The depth of cancer involvement was classified according to the Japanese Classification of Esophageal Carcinoma [29]. Written informed consent was obtained from all patients prior to enrollment.

### Statistical analysis

The index lesion for the study was squamous cell carcinoma, including carcinoma in situ. For the statistical analysis, the histological results of resected specimen served as the reference standard. Evaluation was performed on a per-lesion basis, and the lesion was considered to be the unit of analysis. For patients with more than one lesion, each lesion was considered to be an independent observation for statistical purposes.

The primary outcome variable in this study was the accuracy of endoscopic diagnosis and biopsy diagnosis. The specificity, positive predictive value (PPV), negative predictive value (NPV), and accuracy were calculated as follows: Sensitivity = correctly diagnosed cancers/total cancers; Specificity = correctly diagnosed noncancers/total noncancers; PPV = total cancers/total lesions diagnosed as cancers; NPV = total noncancers/total lesions diagnosed as noncancers; and Accuracy = correctly diagnosed lesions/total lesions.

A noninferiority trial design was chosen to investigate the accuracy of endoscopic diagnosis using magnifying NBI. In a noninferiority trial, a slightly reduced diagnostic accuracy might be accepted if it is balanced by other secondary benefits; in the case of optical biopsy using magnifying NBI, these benefits include less invasiveness, less cost, and real-time results. Noninferiority of endoscopic diagnosis is established when the difference between endoscopic diagnosis and biopsy diagnosis is not smaller than the prespecified noninferiority margin. We chose a noninferiority margin (D) of −10% at the outset of this trial because we considered that this level would balance the clinical efficacy and secondary benefits. Previous studies have reported that the diagnostic accuracy of optical biopsy using magnifying NBI is approximately 90% [23]. Therefore, we hypothesized that optical biopsy diagnosis and histological diagnosis of biopsy specimens would achieve an accuracy of 90%. The study required at least 110 lesions for a 10% threshold of noninferiority and a statistical power of 80% with statistical significance set at p < 0.05. McNemar’s test was used to compare the accuracy of endoscopic diagnosis and biopsy diagnosis. For all analyses, a p value of <0.05 was considered statistically significant.

Subgroup analysis was performed to compare the outcomes among subgroups divided according to lesion size and cancer invasion depth (cancer in situ or invasive cancer) to confirm the consistency of the results.

## Results

### Primary endpoint

**Figure 4:**
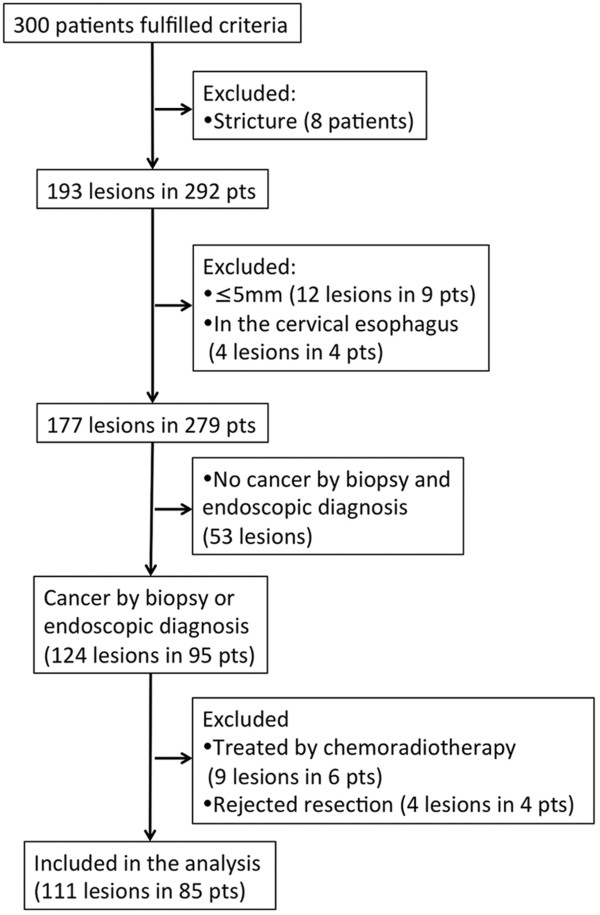
Patient disposition.

Between November 2010 and October 2012, a total of 300 patients who fulfilled our criteria underwent endoscopic examination (Figure 4). A total of 193 lesions were detected in these patients, and 111 lesions in 85 patients were included in the analysis. Of the 111 lesions, 100 lesions were diagnosed as HGIN or cancer by magnifying NBI. Eight lesions were diagnosed as undetermined by magnifying NBI but as cancer by histologic diagnosis of the biopsy specimens. Two lesions were diagnosed as no cancer by magnifying NBI but as cancer by histologic diagnosis of the biopsy specimens. One lesion was diagnosed as no cancer by magnifying NBI and histologic diagnosis of the biopsy specimens but as cancer by iodine staining.

**Figure 5:**
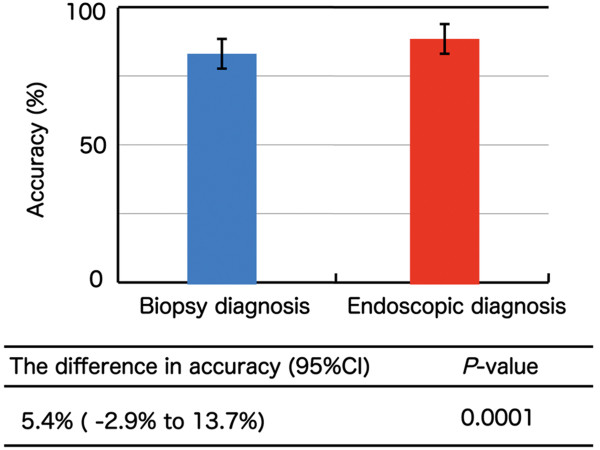
Accuracy of biopsy diagnosis and endoscopic diagnosis in all lesions.

A single biopsy specimen was taken from the lesion in 105 of the 111 lesions, and 2 biopsy specimens were taken from the other 6 lesions. Of the 111 lesions, 78 were invasive cancer, 32 were intraepithelial cancer, and 1 was low-grade intraepithelial neoplasia. The median (range) lesion size was 20 mm (6–100 mm). In total, 23 lesions were located in the upper esophagus, 63 were in the mid-esophagus, and 25 were in the lower esophagus. The accuracy, sensitivity, and specificity of endoscopic diagnosis by magnifying NBI were 91.0% (101/111) (95% CI: 84.1%–95.6%), 90.9% (100/110) (95% CI: 83.9%–95.6%), and 100% (1/1) (95% CI: 2.5%–100.0%), respectively (Table 1). The accuracy, sensitivity, and specificity of histologic diagnosis of biopsy specimens were 85.6% (95/111) (95% CI: 77.7%–91.5%), 86.4% (95/110) (95% CI: 78.5%–92.2%), and 0% (0/1) (95% CI: 0.0%–97.5%), respectively (Table 1). The difference in diagnostic accuracy was 5.4% (95% CI: −2.9%–13.7%). The lower bound of the 95% confidence interval was above the prestated −10%; thus, the primary endpoint was reached and the noninferiority of endoscopic diagnosis by magnifying NBI to histologic diagnosis by biopsy specimen was established (p = 0.0001) (Figure 5).

**Table 1:** Accuracy of endoscopic diagnosis and histologic diagnosis

	**Endoscopic diagnosis**	**Histologic diagnosis**
Sensitivity		
Value (95% CI^†^)	90.9% (83.9–95.6%)	86.4% (78.5–92.2%)
No.lesions	100/110	95/110
Specificity		
Value (95% CI)	100% (2.5–100.0)	0% (0.0–97.5%)
No.lesions	1/1	0/1
Positive predictive value		
Value (95% CI)	100% (96.4–100.0%)	99.0% (94.3–100.0%)
No.lesions	100/100	95/96
Negative predictive value		
Value (95% CI)	9.1% (0.2–-41.3%)	0% (0–21.8%)
No.lesions	1/11	0/15
Accuracy		
Value (95% CI)	91.0% (84.1–95.6%)	85.6% (77.7–91.5%)
No.lesions	101/111	95/111

^†^CI: confidence interval.

### Subgroup analysis

The accuracy of endoscopic diagnosis in lesions ≤10 and >10 mm was 77.8% (21/27) (95% CI: 57.7%–91.4%) and 95.2% (80/84) (95% CI: 88.3%–98.7%), respectively. The accuracy of histologic diagnosis of biopsy specimens in lesions ≤10 and >10 mm was 74.1% (20/27) (95% CI: 53.7%–88.9%) and 89.3% (75/84) (95% CI: 80.6%–95.0%), respectively. The difference in the diagnostic accuracy was 3.7% (95% CI: −20.4%–27.8%, p = 0.13) in lesions ≤10 mm and 6.0% (95% CI: −1.7%–13.7%, p < 0.0001) in lesions >10 mm.

The accuracy of endoscopic diagnosis in epithelial lesions and invasive cancers was 81.8% (27/33) (95% CI: 64.5%–93.0%) and 94.9% (74/78) (95% CI: 87.4%–98.6%), respectively. The accuracy of histologic diagnosis of biopsy specimens in epithelial lesions and invasive cancers was 87.9% (29/33) (95% CI: 71.8%–96.6%) and 84.6% (66/78) (95% CI: 74.7%–91.8%), respectively. The difference in the diagnostic accuracy was −6.1% (95% CI: −24.8%–12.7%, p = 0.34) in epithelial lesions and 10.3% (95% CI: 1.6%–19.0%, p < 0.0001) in invasive cancers. With the exception of intraepithelial lesions, endoscopic diagnosis showed results preferable to those of histologic diagnosis of biopsy specimens, and the consistency of the results was confirmed in the subgroup analyses.

### Retrospective analysis of misdiagnosis

**Figure 6:**
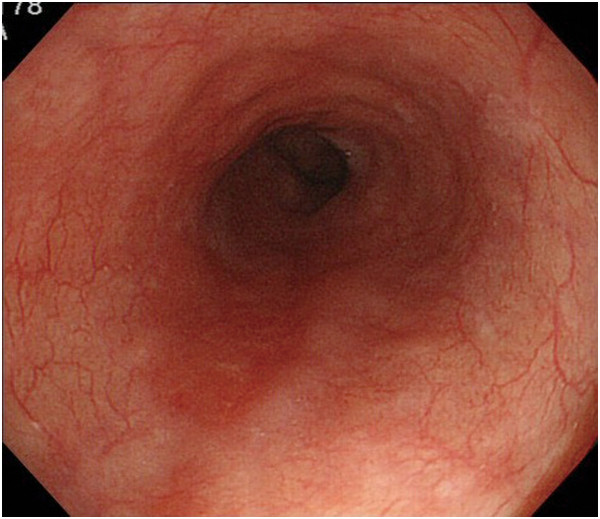
White-light imaging shows a reddish lesion 20 mm in diameter on the posterior wall of the lower esophagus.

**Figure 7:**
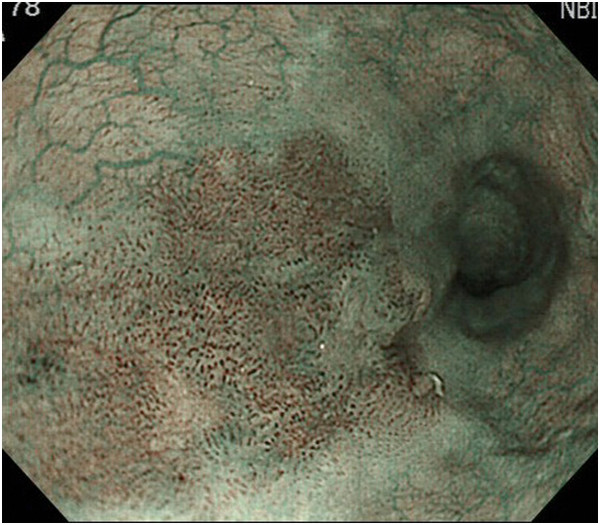
Narrow-band imaging shows well-demarcated brownish change of the epithelium and the presence of scattered brown dots.

**Figure 8:**
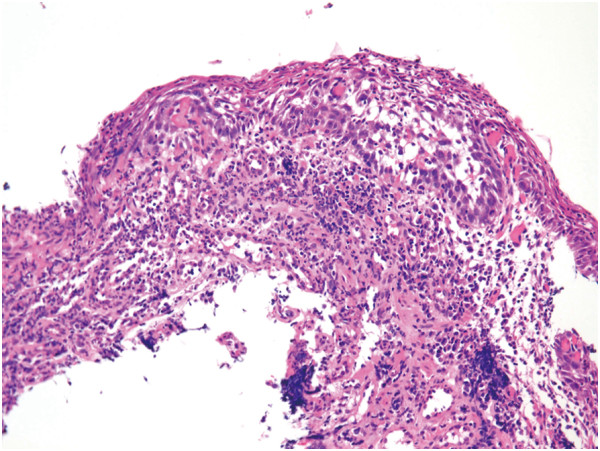
Biopsy diagnosis of cancer was not made because of inflammation.

**Figure 9:**
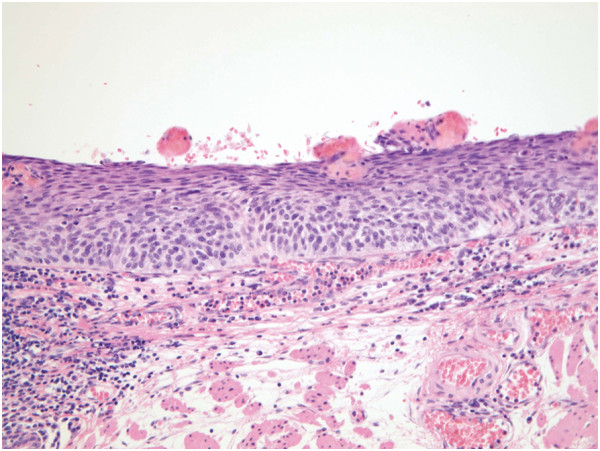
Histologic diagnosis of a resected specimen was cancer in situ.

Retrospective analysis of 15 cancers misdiagnosed as no cancer by biopsy was performed (Figure 6,7,8 and 9). Cytological abnormalities were confirmed in 13 of the 15 lesions. Of these 13 lesions with cytological abnormalities, 11 were misdiagnosed because the atypia was weak, and 2 were misdiagnosed because of concomitant inflammation. Another two lesions were probably misdiagnosed due to sampling error because no cytological abnormalities were observed in the biopsy specimens.

Retrospective analysis of 10 cancers endoscopically misdiagnosed as undetermined or no cancer was performed. Of these 10 lesions, vascular change was not obvious in 4, brownish change of the epithelium was not obvious in 2, neither of these changes was obvious in 3, and the mucosal surface was not observed because of extensive keratosis in 1.

## Discussion

The accuracy of endoscopic diagnosis and biopsy diagnosis was 91.0% (101/111) (95% CI: 84.1%–95.6%) and 85.6% (95/111) (95% CI: 77.7%–91.5%). The difference in diagnostic accuracy was 5.4% (95% CI: −3.9%–13.8%), and the noninferiority of endoscopic diagnosis by magnifying NBI to histologic diagnosis by biopsy was established (p < 0.001). Our study is unique because lesions diagnosed as cancer by endoscopic and biopsy examination were included in the study. This study is the first to show the accuracy of endoscopic diagnosis compared with biopsy diagnosis using a noninferiority trial design.

Diagnosis of gastrointestinal cancers is based on the detection of suspicious lesions and histological evaluation of biopsy specimens taken from these suspicious lesions. Although biopsy diagnosis serves as the gold standard pretreatment diagnosis, it is associated with high false-negative rates [8,9]. False-negative biopsy diagnosis may occur secondary to error in the specimen retrieval process. However, in this study, all biopsies were taken from the lesions, which was confirmed by recorded pictures. Focally distributed cancer can be missed by biopsy, even if the specimen is taken from the lesion. Considering that only a small part can be examined by biopsy, this sampling error is a basic limitation of biopsy rather than technical error.

A single biopsy specimen was taken from the lesion in 105 of 111 lesions and from all 15 lesions with a false-negative biopsy diagnosis. Multiple biopsies may improve the accuracy of biopsy diagnosis. In previous reports, 3 to 4 biopsies [10], 4 to 6 biopsies [12], and 10 biopsies [13] are recommended to obtain high diagnostic accuracy. Multiple biopsies are acceptable for patients with advanced cancers that will be treated by surgical resection. However, multiple biopsies may cause problems for patients with early cancers because submucosal fibrosis caused by multiple biopsies sometimes interferes with the endoscopic resection process. Considering the potential disadvantage of multiple biopsies, the importance of endoscopic diagnosis rather than multiple biopsies for superficial lesions should be emphasized.

In recent years, several new endoscopic imaging techniques have been developed that may improve the detection and diagnosis of early esophageal cancer. NBI is a novel imaging technique that enhances the visualization of mucosal microstructures and microvessels. The addition of the magnification component has further allowed visualization of very minute mucosal details and hence histologic prediction in real time. Previous studies of NBI and magnification have shown a high diagnostic accuracy for esophageal squamous cell carcinoma, raising the expectation of optical biopsy in the clinical setting. However, the accuracy of endoscopic diagnosis has not been directly compared with that of other modalities. Before it can be regarded as a useful modality for diagnosis of cancer, it should be compared with the current standard modality of biopsy diagnosis. Therefore, we conducted the current study and showed the noninferiority of endoscopic diagnosis compared with the accuracy of biopsy diagnosis.

This study was conducted based on the assumption that biopsy diagnosis and endoscopic diagnosis are tested modalities and that only histologic diagnosis of resected specimens can be regarded as the reference standard. Based on these assumptions, unresected lesions were not included in the analysis because the reference standard of the resected specimens was not obtained in these lesions. However, even if those lesions were included as noncancer, the noninferiority of endoscopic diagnosis was established.

A noninferiority trial design was chosen to investigate the utility of endoscopic diagnosis compared with biopsy diagnosis. In a noninferiority trial, a slightly reduced clinical efficacy might be accepted if it is balanced by other secondary benefits; in the case of endoscopic diagnosis, these benefits include less invasiveness, lower cost, and real-time results. We chose a stringent and conservative noninferiority margin (Δ) of 10% [30] and showed the noninferiority of endoscopic diagnosis compared with biopsy diagnosis. In this study, lesions with obvious cytological abnormalities were diagnosed as cancer even when they were confined to the lower half of the squamous epithelium. There are some issues regarding the diagnosis of these lesions. In Western countries, these lesions are diagnosed as low-grade intraepithelial neoplasia and are not diagnosed as cancer. Therefore, we conducted subgroup analysis of invasive cancers. Noninferiority of the endoscopic diagnosis to biopsy diagnosis was also confirmed in this subgroup. Subgroup analyses were also performed among subgroups divided according to lesion size and intraepithelial lesions. The accuracy of endoscopic diagnosis was comparable with that of biopsy diagnosis in all subgroups, thus enhancing the reliability of the study conclusions.

This study is limited because all lesions were not confirmed by the reference standard of resected specimens. Considering the risk of endoscopic resection or surgical resection, resecting lesions diagnosed as noncancer by endoscopy or biopsy would not be acceptable.

## Conclusions

This study showed that the accuracy of endoscopic diagnosis is comparable with that of biopsy diagnosis. This finding may facilitate the practical use of endoscopic optical diagnosis.

## Competing interests

The authors declare that they have no competing interst.

## Authors’ contributions

RI, KN, NU, HK, TO, YTa, and SI planned the study. HK, RI, TO, YTo, and SI conducted the study. KN, HK, RI, TO, and TM collected the data. KN, HK, RI, TO, TY, KA, NM, TI, MF, SY, NH, KH, HI, MT and TM interpreted the data. KN and RI drafted the manuscript. All authors read and approved the final draft.

## Pre-publication history

The pre-publication history for this paper can be accessed here:

http://www.biomedcentral.com/1471-230X/14/141/prepub
